# Selective Laser Melting of Maraging Steel Using Synchronized Three-Spot Scanning Strategies

**DOI:** 10.3390/ma14081905

**Published:** 2021-04-11

**Authors:** Chung-Wei Cheng, Wei-You Jhang Jian, Bhargav Prasad Reddy Makala

**Affiliations:** 1 Department of Mechanical Engineering, National Yang Ming Chiao Tung University, No. 1001, Ta Hsueh Road, Hsinchu 300, Taiwan; kenasparagus@gmail.com (W.-Y.J.J.); bhargav77.me06g@nctu.edu.tw (B.P.R.M.); 2 Department of Mechanical Engineering, National Chiao Tung University, No. 1001, Ta Hsueh Road, Hsinchu 300, Taiwan

**Keywords:** selective laser melting, laser powder bed fusion, multi-spot, scanning strategy

## Abstract

The selective laser melting (SLM) process, a kind of metal additive manufacturing method, can produce parts with complex geometries that cannot be easily manufactured using material removal processes. With increasing industrial applications, there are still issues such as part quality and productivity that need to be resolved. In this study, maraging steel parts fabricated by synchronized three-spot scanning strategies, i.e., lateral spatial (LS) and spatial inline (SiL), are firstly presented. The LS and SiL represent the three-spot offset direction is perpendicular and parallel to the scanning direction, respectively. A laboratory SLM machine equipped with a fiber laser and three-spot module is used to fabricate the maraging steel parts with two scanning strategies, i.e., LS and SiL. The influence of these scanning strategies on the surface roughness, relative density, hardness, molten pool shapes, and microstructures are investigated. The relative density (~99.02%) and surface hardness (~34.0 HRC) are experimentally found to be higher than the SiL by the LS scanning strategy.

## 1. Introduction

Selective laser melting (SLM) or laser powder bed fusion is a unique additive manufacturing technology for producing parts with complex geometry and mechanical properties comparable to bulk materials [1]. Metallic powders are melted layer-by-layer and solidified to metal parts by selectively scanning the focused laser beams. Childs et al. [2] studied the influence of laser power and scanning speed on the solidification structure, and the shape of the molten track was classified into different types: continuous and flat-topped, continuous and rounded, occasionally broken, balled, and partially melted. Yadroitsev et al. [3] studied the influence of scanning speed on the remelt depth. A higher thermal penetration depth was achieved with low scanning speed, which enhances the bonding strength between the fabricated layer and the previous layer.

However, a higher temperature gradient usually occurs in the SLM process. The high-temperature gradient around the laser irradiation area usually leads to higher thermal stress during the process, resulting in residual stress, part deformation, and crack generation [4,5]. Process parameters such as laser power, scanning speed, or scanning strategy will affect the temperature gradient generated in the SLM process. The scanning strategy can effectively disperse thermal stress, thereby reducing deformation and residual stress [6]. Matsumoto et al. [7] proposed that the laser scanning trajectory could be shortened, and the scanning area could be divided into grids to avoid residual thermal stress accumulation. Kruth et al. [8] experimentally verified that the island scanning pattern could effectively suppress excessive thermal deformation. Furthermore, post-processing methods, such as hot isostatic pressing and hot forging [9], can be used to improve product characteristics.

Recently, novel multi-beam strategies on the laboratory SLM machine used to improve the processing speed and reduce residual stress have been presented [10,11,12,13]. The extra added beam affects the temperature gradient in the molten pool area, such as preheating or controlling the cooling rate. Wilkes et al. [10] used a CO_2_ laser to preheat the ceramic powders to 1800 °C. Then a fiber laser was used to selectively melt the preheated powders, producing ceramic components with approximately 100% density. Heeling and Wegener [11] used a two-beam strategy; one laser beam was a melting beam (main beam), and the other was a defocused beam (pre- or postheating beam). The two-beam offset was parallel to the scanning direction. The microstructure characteristics of the built stainless steel parts using different relative positions and offsets of the two laser beams were investigated. Tsai et al. [12] used a three-beam strategy, and the beam offset was perpendicular to the scanning direction. The results using the synchronized three-beam method demonstrated an improvement in layer surface roughness. Moreover, the scanning time decreased by 38.1% compared to the single-beam method. Zhang et al. [13] used dual-laser sources with offset direction of the two focus beams perpendicular to the scanning direction. Periodic structures can be made by synchronously scanning the two-spot. However, in [12,13], only the single-layer structures were shown, and the 3D structures were not presented.

In this study, a laboratory SLM machine equipped with a fiber laser and three-spot module was used to build the maraging steel parts. Cubic parts fabricated by two scanning strategies, i.e., lateral spatial (LS) and spatial inline (SiL), are presented. The LS and SiL represent the three-spot offset direction is perpendicular and parallel to the scanning direction, respectively. The influence of the two scanning strategies on the surface roughness, relative density, hardness, molten pool shapes, and microstructures are evaluated.

## 2. Experiment

**Figure 1:**
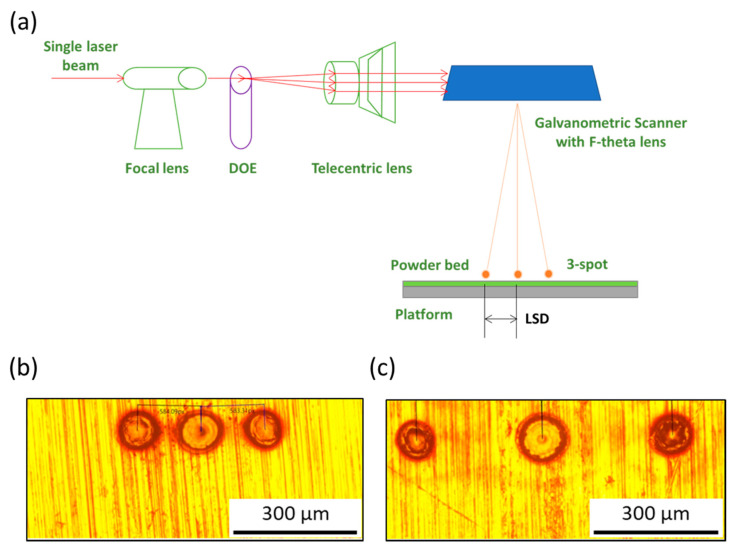
(**a**) Schematic illustration of a laboratory SLM machine with a three-spot module; (**b**,**c**) images of different LSD irradiated on the S45C material.

A laboratory SLM machine (as illustrated in Figure 1a) with a fiber laser and a three-spot module was used to build the parts. A fiber laser (YLR-200-AC-Y14, IPG Photonics, Massachusetts, USA) with a wavelength of 1070 nm and a laser power of 200 W was used. A three-spot module equipped with a focal lens of focal length 100 mm, a diffractive optical element (DOE, DFT-3L, Montreal, Canada) of 1 × 3 spots, a telecentric lens with a focal length of 163 mm, and a galvanometric scanner (intelliSCAN 20, Scanlab, Puchheim, Germany) with an F-theta lens with a focal length of 210 mm. The split efficiency of the three spots after DOE was determined to be about 28.5%, 28.0%, 27.0%, and the angular separation between the two adjacent beams was determined to be 2.36°. A focal lens was used to adjust the divergence angle of the individual laser beams. The diameter of the three focal spots on the powder bed was determined to be about 60 μm.

The distance between the focused beams on the powder bed, i.e., lateral spatial distance (LSD), could be adjusted by changing the relative position of the DOE on the optical path, because the telecentric lens would change divergence angle of the three laser beams through the DOE, resulting in the distance between the three focused beams on the working plane changing. Figure 1b,c presents examples of two different LSD irradiated on the bulk material (S45C), about 155 μm and 310 μm, respectively. If the DOE moves toward the telecentric lens, the LSD on the powder bed decreases, as shown in Figure 1b. If the DOE position moves toward the focal lens, the LSD on the powder bed increases, as shown in Figure 1c.

**Figure 2:**
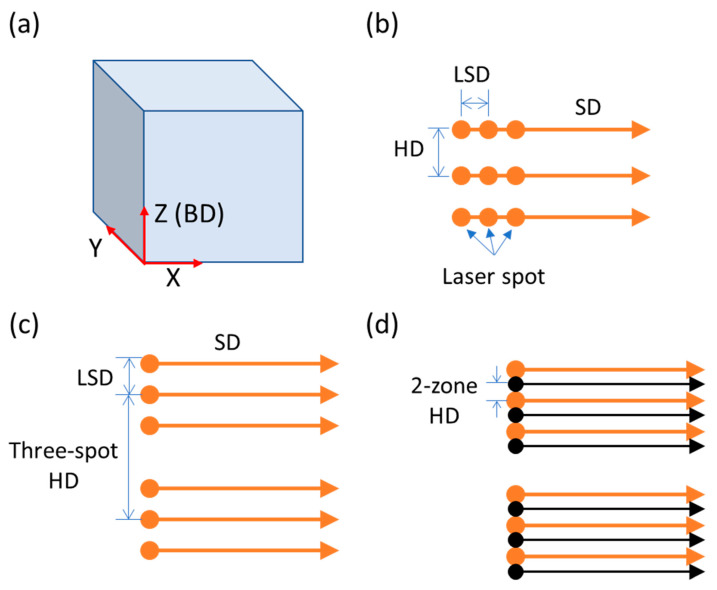
Schematic diagram of (**a**) built sample with coordinate axes; (**b**) SiL scanning and (**c**,**d**) LS scanning strategies in the XY plane.

Figure 2 presents a schematic illustration of synchronized three-spot scanning using the SiL (spatial inline) and LS (lateral spatial) strategies on a powder bed. In Figure 2a, the building direction (BD) is along the Z direction, and the powder bed is in the XY plane. For the SiL scanning strategy, as shown in Figure 2b, the filled circle represents the laser spots, the three-spot offset distance is parallel to the scanning direction (SD). The distance between adjacent scanning paths is defined as hatch distance (HD). Please note that in the SiL scanning strategy, the current DOE splitting efficiency for the three spots is similar, resulting in the power density of each laser spot is similar. For the LS scanning strategy, as shown in Figure 2c,d, the three-spot offset distance is perpendicular to the SD, and the three-spot HD (three-spot hatch distance) is defined as the distance between two three-spot scanning paths. In Figure 2d, the second path (black color) of the three-spot process is scanned in the same direction again using the offset (2-zone HD) from the first scanning path (orange color). In both scanning strategies, the SD was rotated by 90° after every single layer.

In the SLM process, maraging steel powder (EOS MS1, EOS GmbH, Krailling, Germany) with a layer thickness of 40 μm was deposited on a base plate (S45C) substrate. The main material composition of maraging steel powder is Fe (balance), Ni (~18 wt.%), Co (~9 wt.%), Mo (~5 wt.%). The SLM process was performed in a chamber filled with protective gas (argon). It had a uniform flow rate on the powder surface, and the oxygen concentration was controlled to less than 750 ppm to avoid oxidation during processing. After the SLM processing, the fabricated sample was removed from the base plate by wire cutting. The relative density of the fabricated parts was measured using the Archimedes principle by densimeter (MGR-120, MatsuHaku, Taichung City, Taiwan) and compared to the EOS MS1 density of about 8.1 g/cm^3^. The surface roughness and hardness were measured using surface roughness measurer (SJ-210, Mitutoyo, Kanagawa, Japan) and hardness test equipment (LC-200R, Future–Tech, Kanagawa, Japan). A scanning electron microscope (SEM, SU-8010, Hitachi, Tokyo, Japan) equipped with an electron back-scattered diffraction (EBSD) detector was used for detailed microstructural characterization. For microstructure investigation, the samples were wire cut from the base plate and sectioned parallel to the building direction. The cross-section SEM measurement samples were mechanically polished and chemically etched in 5% HNO_3_ for 20 s.

The EBSD measurement sample was ground with sandpapers (#80~#2500) to a smooth surface, polished with 1 μm alumina powder for 10 minutes, and 0.05 μm alumina powder for 10 minutes. Finally, a non-crystallizing colloidal silica polishing suspension (particle size 0.02 μm) was used to polish for 10 minutes to eliminate the stress layer. The microstructure orientation and phase formation were analyzed by EBSD for an area of 50 × 50 μm^2^ with a 0.25 μm step size.

## 3. Results and Discussion 

### 3.1. Single-Layer Process

**Figure 3:**
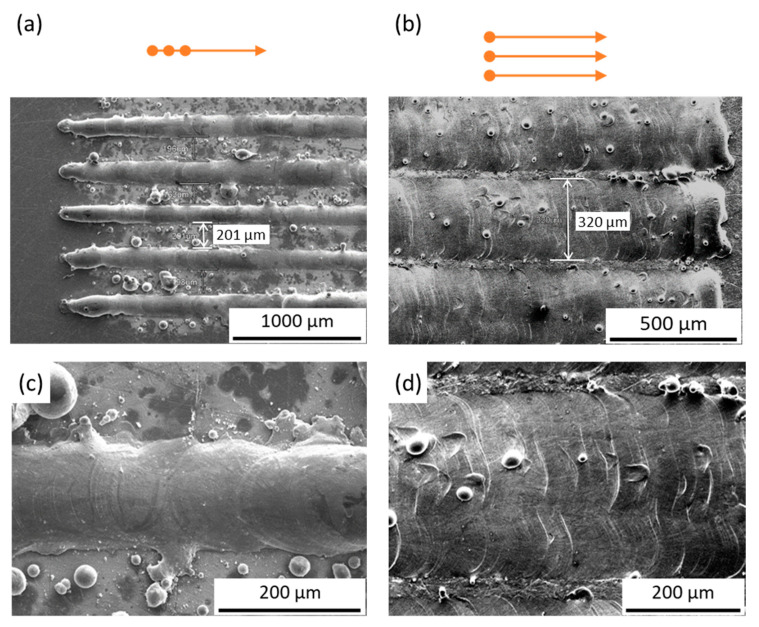
SEM images of the single-layer molten track fabricated by (**a**) SiL and (**b**) LS scanning strategies. (**c**,**d**) Magnified images of (**a**,**b**), respectively.

Figure 3 presents the surface morphology of the single-layer molten tracks on the baseplate fabricated by the three-spot with SiL and LS scanning strategies, respectively. The total laser power was 145 W, scanning speed was 70 mm/s, LSD was 250 μm, HD was 350 μm, three-spot HD was 350 μm, and 2-zone HD was 175 μm. In Figure 3a, an individual molten track was produced on the substrate. In Figure 3b, the merged molten tracks were formed on the substrate fabricated by LS with the 2-zone scanning strategy. Figure 3c shows the single molten track fabricated by SiL, and the width is about 170 μm. Figure 3d shows the molten track fabricated by LS, and the width is about 320 μm. Because the three-spot LSD distance is perpendicular to the SD (see Figure 2c), the width of the molten track by LS is larger than SiL. Please note that to observe the individual molten track, higher values of HD, three-spot HD, and 2-zone HD were used. These parameters need to be adjusted (see Section 3.2) to get full density parts. 

### 3.2. Cubic Parts

**Figure 4:**
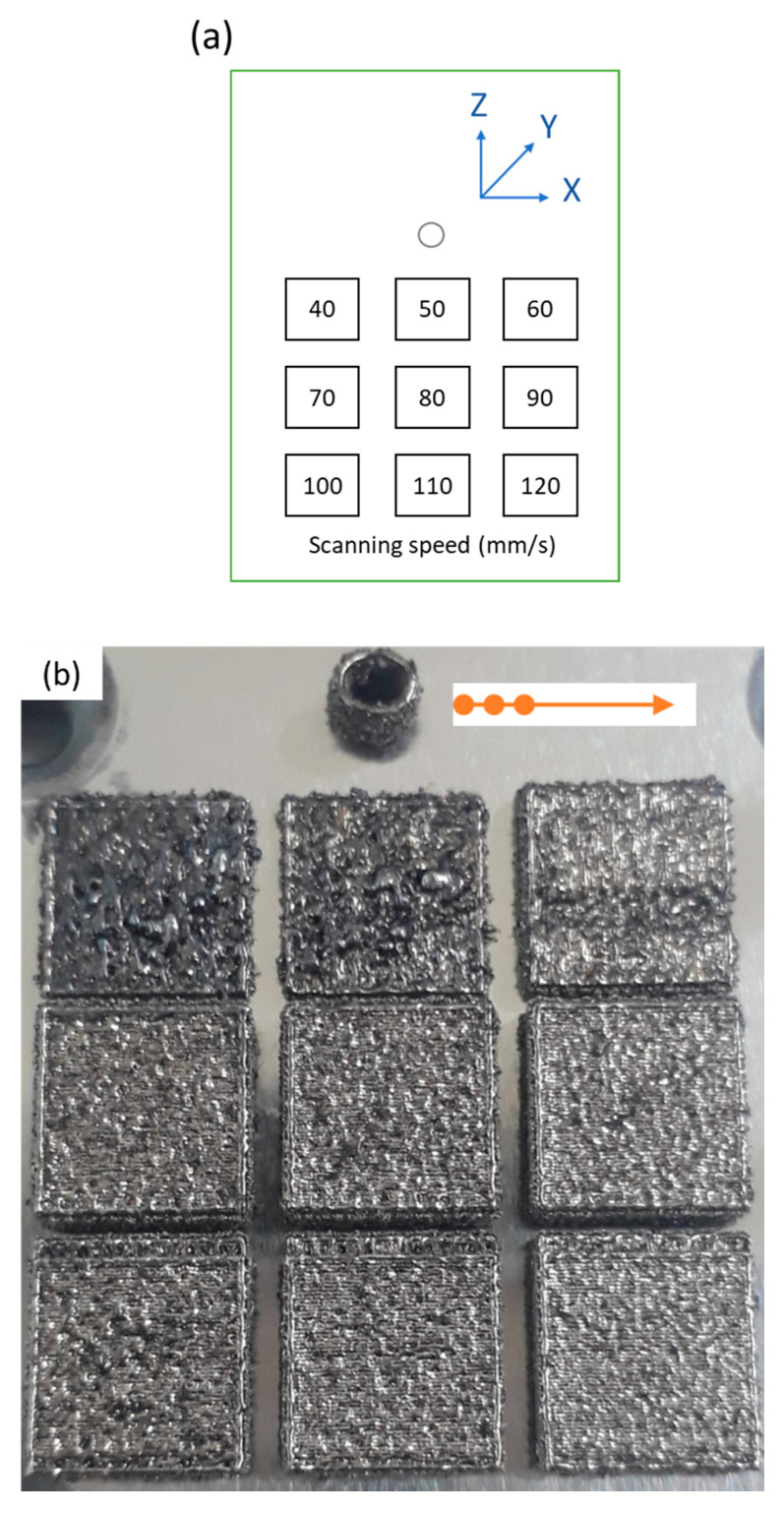

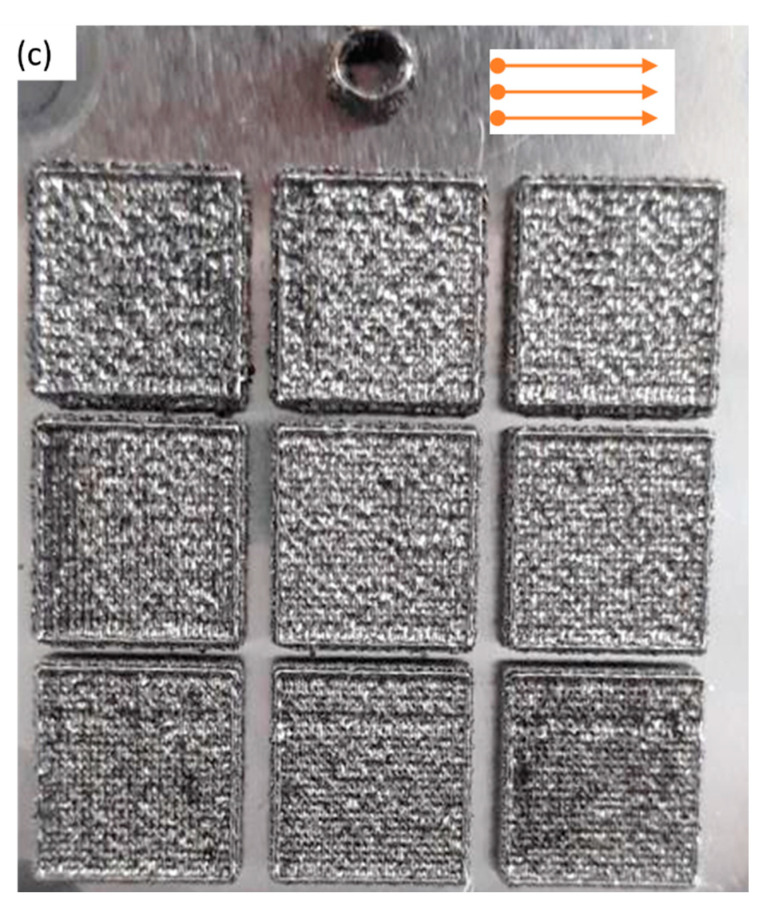
Photographs of cubic parts fabricated by (**a**) design parameters with scanning speed 40~120 mm/s; (**b**) SiL and (**c**) LS scanning strategy.

Figure 4 shows the parts with a size of 7 × 7 × 2 mm^3^ fabricated using the SiL and LS strategies, respectively. The scanning speed was in the range of 40–120 mm/s, total laser power was 145 W, LSD was 250 μm, HD was 125 μm, three-spot HD was 250 μm, and 2-zone HD was 125 μm. Each laser spot power was about 48.3 W, and the power density for each laser spot was about 3.42 × 10^6^ W/cm^2^ (spot diameter ~60 μm). This exceeds the melting threshold of maraging steel powder, so that the individual laser spot can produce melting [14]. In the SiL case, as shown in Figure 4b, the scanning speed of 70–120 mm/s can produce uniform structures with an average roughness (Ra) of around 8.3 ± 0.7 µm. However, due to the low scanning speed (40–60 mm/s) and the three-spot along the scanning direction, more excessive irradiation energy results in spatter formation during the SLM process, and high surface roughness on the fabricated parts occurred.

In the SL case, as shown in Figure 4c, it can be seen that the surface roughness does not change much, and the average Ra was around 9.3 ± 0.6 µm. It can be found that the average Ra produced by the two scanning methods is similar, mainly because of the different re-melting effects: (1) SiL: the second and third laser spot followed by the first spot scanning path; (2) SL: the second path of the three-spot scanned in the same direction again using the offset from the first scanning path.

**Figure 5:**
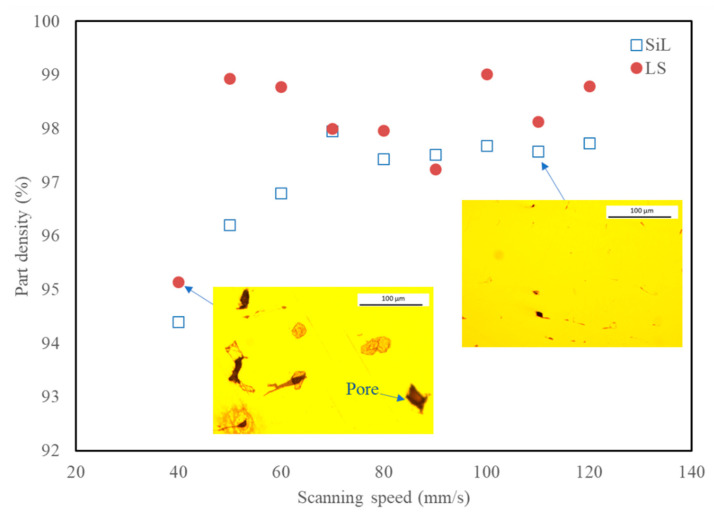
Relative density of maraging steel parts fabricated by SiL and LS scanning strategies.

**Figure 6:**
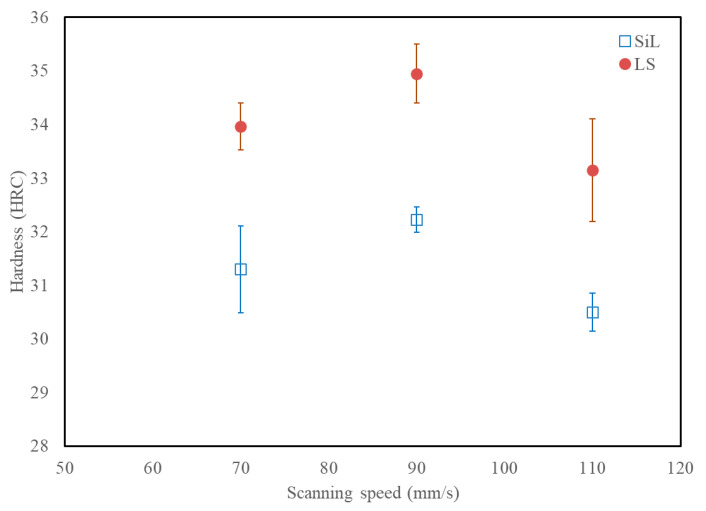
Surface hardness of maraging steel parts fabricated by SiL and LS scanning strategies.

Figure 5 shows the relative density of these samples. It can be observed that cubic parts fabricated by SL have a better density than SiL. The average relative density of the LS strategy achieved 98.4% for scanning speeds 50–120 mm/s. The maximum relative density of the LS strategy reached 99.02% at a scanning speed of 100 mm/s. However, at the low scanning speed (40 mm/s), more excessive irradiation energy results in spatter formation and leads to a few pores, as shown in the inset OM image. Figure 6 shows the surface hardness of these samples. The average surface hardness for the LS specimens fabricated at scanning speed 70–110 mm/s is about 34.0 HRC. It can be observed that cubic parts fabricated by SL have a better surface hardness than SiL. Please note that SL can have a better density and surface hardness than SiL, mainly because of the different characteristics of the molten pool and microstructures produced during the SLM process, as will be further explained in Section 3.3.

### 3.3. Microstructure Analysis

**Figure 7:**
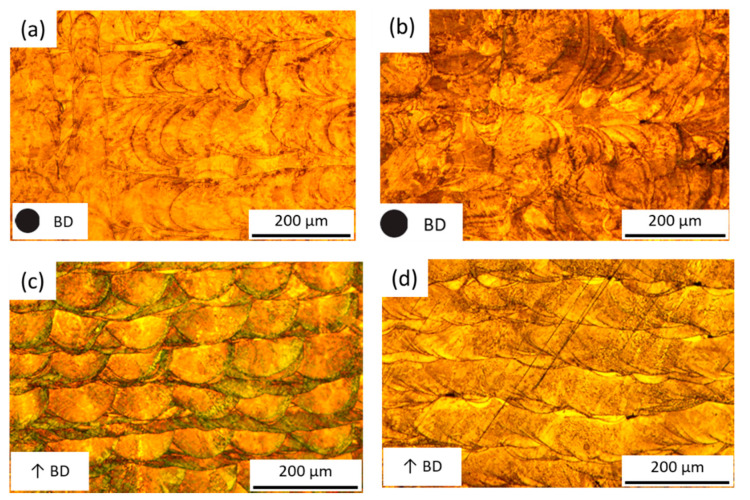
OM images of the microstructure fabricated by (**a**,**c**) SiL and (**b**,**d**) LS with scanning speed 110 mm/s; (**a**,**b**) top-view (XY) and (**c**,**d**) cross-sectional (XZ) view.

**Figure 8:**
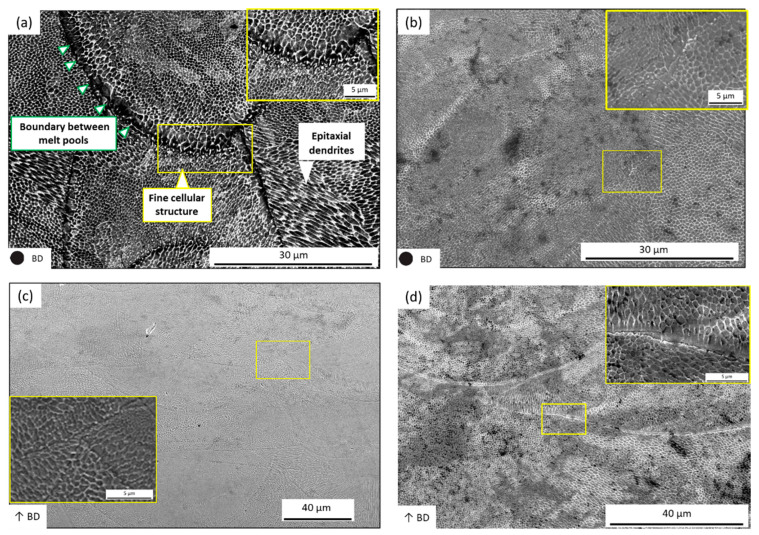
SEM images of the structures by (**a**,**c**) SiL and (**b**,**d**) LS with scanning speed 110 mm/s; (**a**,**b**) top-view (XY) and (**c**,**d**) cross-sectional (XZ) view. The inset shows the zoomed image.

Figure 7 and Figure 8 show the top view (XY plane) and cross-sectional view (XZ plane) microstructure optical micrograph (OM) and SEM images of the cubic parts fabricated by SiL and LS scanning strategy with scanning speed 110 mm/s, respectively. As shown in Figure 7c,d, the shape and size of the molten pools generated by these two scanning methods are different. In Figure 7c, the SiL sample shows a hemispherical molten pool morphology with depth and width of around 50 μm and 120 μm, respectively. Maraging steel parts were built using the SLM process with a single Gaussian beam and hexagonal grid scanning pattern presented in [15], and the molten pool shape was also similar to hemispherical. In Figure 7d, the LS sample shows a long semi-elliptical molten pool morphology with a depth and width of around 50 μm and 450 μm, respectively. Because the three-spot LSD distance is perpendicular to the SD (see Figure 2c,d), the width of the molten pool by LS is larger than SiL. Hastelloy alloy parts were built using the SLM process with a single laser beam with broad beam diameter presented in [16], and the shape of the molten pool was also similar to the semi-elliptical shape.

Figure 8a shows the top-view (XY plane) microstructure of the maraging steel parts fabricated using the SiL scanning strategy, which is mainly divided into the fine cellular structure (size is about 1 μm) and epitaxial dendrites. The cellular structure is composed of tiny loops, and the structure of the epitaxial dendrite is composed of thin and long loops. The appearance of epitaxial dendrites is primarily due to rapid cooling to form martensite, and the microstructure formations are related to the melt-region heat flux direction around the molten pool. Please note that these microstructures can also be observed in the LS case (see Figure 8b) and cross-sectional images for the SiL and LS cases (see Figure 8c,d). Therefore, it is speculated that the cross-section of the structure of the epitaxial dendrite is the cellular structure. Because the direction of the cut plane is different, two different structures are observed.

**Figure 9:**
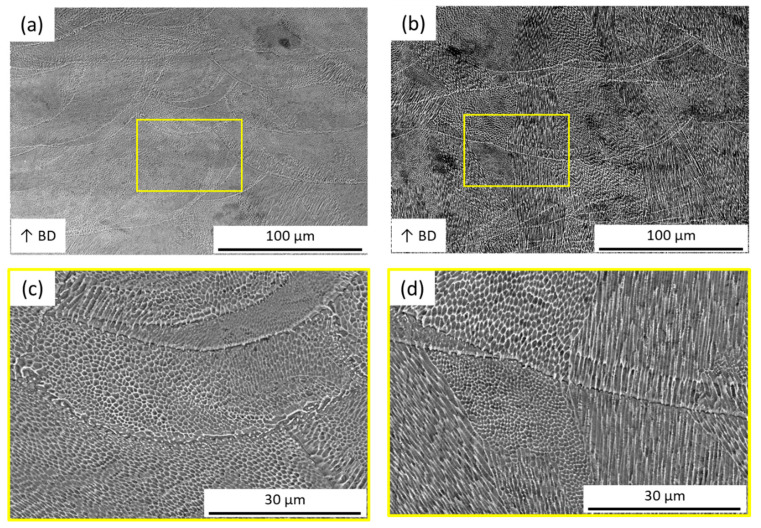
Cross-sectional (XZ) SEM images of the microstructures with the LS scanning strategy and scanning speed of (**a**) 70 mm/s and (**b**) 110 mm/s; (**c**,**d**) magnified image of the yellow and red block shown in (**a**,**b**), respectively.

**Figure 10:**
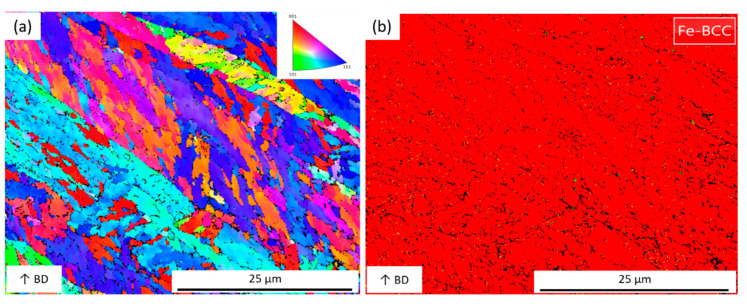
(**a**) orientation color map and (**b**) phase map of Figure 9b.

The cross-sectional (XZ plane) microstructure images by LS scanning strategy with scanning speed 70 mm/s and 110 mm/s are compared in Figure 9, respectively. At low scanning speed, the molten pool shape is more rounded (see Figure 9a). The slow scanning speed leans to a higher thermal penetration depth, resulting in a rounder molten shape. When the scanning speed increases, the thermal penetration depth decreased, and the molten pool gradually becomes smooth (see Figure 9b). In Figure 9d, the microstructure directions are more diverse, and epitaxial dendrite structures are formed. The higher the scanning speed, the faster the cooling rate, and the liquid maraging steel quickly becomes solid. Figure 10 shows the orientation color map and phase map of an area in Figure 9b. The orientation color map presents many grains with different crystallographic orientations to the build direction are presented, and the Fe-BCC is the majority.

## 4. Conclusions

This study reported maraging steel parts fabricated by a three-spot SLM system with two novel scanning strategies: lateral spatial (LS) and spatial inline (SiL). The LS and SiL are defined as the three-spot offset perpendicular or parallel to the scanning direction. The experimental results show the influence of the two scanning strategies on the maraging steel part’s surface roughness, relative density, hardness, molten pool shape, and microstructures. The main results are as follows:

1. The average surface roughness Ra produced by the two scanning methods is similar, i.e., around 8.3~9.3 µm, mainly because of the different re-melting effects: (1) SiL: the second and third laser spot followed by the first spot scanning path; (2) SL: the second path of the three-spot scanned in the same direction again using the offset from the first scanning path.
2. The relative density and surface hardness produced by the LS scanning strategy is higher than the SiL because the SL can produce fewer pores and finer microstructures inside the specimen. The maximum relative density and average surface hardness by the LS scanning are about 99.02% and 34.0 HRC.
3. The morphology of the molten pools generated by the two scanning methods is different, i.e., the molten pool of LS and SiL samples show long semi-elliptical and hemispherical shapes, respectively. The LS scanning strategy resulted in wide and flat molten pools with fine cellular and dendrites microstructures.
4. At present, the split efficiency of the three spots after DOE is similar. In the future, the new DOE design can change the splitting efficiency. For example, the second point is the main beam, and the first and third spots are the pre- and post-heating beam (power is smaller than the main beam). It is expected that the microstructure characteristics by SiL scanning strategy can be further improved.
